# FAM201A Promotes Cervical Cancer Progression and Metastasis through miR-1271-5p/Flotillin-1 Axis Targeting-Induced Wnt/*β*-Catenin Pathway

**DOI:** 10.1155/2022/1123839

**Published:** 2022-10-03

**Authors:** Yuehong Wang, Zhilian Wang, Keyan Cheng, Qirong Hao

**Affiliations:** Department of Obstetrics and Gynecology, Second Hospital of Shanxi Medical University, China

## Abstract

This study investigated the role of the family with sequence similarity 201-member A (FAM201A), as previously reported oncogenic, in cervical cancer (CC). FAM201A expression in CC was analyzed through bioinformatics analyses, and its distribution in CC tissues/cells was determined by *in situ* hybridization. CC cells were transfected/cotransfected with FAM201A/flotillin-1 (FLOT1) overexpression plasmids and miR-1271-5p mimics, followed by functional analysis on viability, migration and invasion. Pearson's correlation tests were performed to analyze the correlation between FAM201A and miR-1271-5p in CC tissues. The targeting relationship between miR-1271-5p and FLOT1 was confirmed by dual-luciferase reporter assay. The expressions of FAM201A, miR-1271-5p, FLOT1, matrix metalloproteinases (MMP)-9, MMP-2, E-cadherin, N-cadherin, and the Wnt/*β*-catenin pathway-related molecules (Wnt1, *β*-catenin and p-*β*-catenin) in CC cells or tissues were assessed by quantitative reverse transcription polymerase chain reaction (qRT-PCR) and/or western blot. The results showed that FAM201A was abundantly expressed and miR-1271-5p expression was downregulated in CC. FAM201A was enriched in CC cell cytoplasm and negatively correlated with miR-1271-5p in CC tissues. FAM201A overexpression enhanced the cell viability, migration, invasion, and tumorigenesis of CC *in vivo* and increased FLOT1 expression. These trends were all reversed by upregulating miR-1271-5p, which induced opposite effects to FAM201A overexpression. MiR-1271-5p upregulation depleted the levels of MMP-9, MMP-2, N-cadherin, and the Wnt/*β*-catenin pathway-related molecules and upregulated E-cadherin expression. FLOT1 was a direct target of miR-1271-5p. FLOT1 overexpression induced effects contrary to the upregulation of miR-1271-5p and abolished miR-1271-5p upregulation-induced effects in CC cells. Overall, this study showed that FAM201A promoted cervical cancer progression and metastasis by targeting the miR-1271-5p/FLOT1 axis-induced Wnt/*β*-catenin pathway.

## 1. Introduction

Cervical cancer (CC) is one of the most common malignant tumors affecting women worldwide, second to breast cancer [1], and is the leading cause of cancer-related mortality in some developing countries [2]. The progression of CC is featured as a multistage and multistep process involving the activation of proto-oncogenes and (or) inhibiting tumor-suppressive genes [3]. Currently, the antitumor treatment for CC remains less effective owing to its late-appearing symptom, leading to unsuccessful disease diagnoses and advanced-stage disease by the time of diagnosis [4]. It is reported that the five-year survival rate for metastatic CC patients is 16.5%, compared to 91.5% for localized CC patients [5]. Therefore, metastasis is accountable for most unfavorable prognoses, recurrence and high morbidity of CC [6].

Long noncoding RNAs (lncRNAs), a type of transcripts constituted by over 200 nucleotides with no translation ability, have emerged as pivotal regulators for the carcinogenesis and progression of cancers, including CC [6]. Epithelial-mesenchymal transition (EMT), a highly conserved trans-differentiation program considered the major driver of cancer progression, is reported to facilitate metastasis of cancer cells by promoting migration and invasion and conferring an apoptosis-resistant property [7]. By directly or indirectly reversing EMT, lncRNAs can repress tumorigenesis, cancer progression, and metastasis, demonstrating their therapeutic potential [8]. The family with sequence similarity 201-member A (FAM201A) is a long nonprotein coding RNA derived from an open reading frame (ORF)-lacking RNA transcripts transcribed from a 2.9 Kbp-long gene that is located in genomic 9p13.1 [9]. Several studies exploring anticancer strategies have revealed the involvement of FAM201A in inducing carcinogenesis and promoting the progression of triple-negative breast cancer (TNBC) [10], lung squamous cell cancer (LSCC) [11], and lung adenocarcinoma (LUAD) [12]. Additionally, highly expressed FAM201A was reported to provoke short-term radio-resistance, leading to inferior survival in patients with esophageal squamous cell cancer [13] and nonsmall-cell lung cancer [14]. However, little is known about the biological roles and clinical significance of FAM201A in CC.

Interactive analyses have identified FAM201A as a key regulator in cancer progression in a lncRNA-miRNA-mRNA competing endogenous RNA (ceRNA) network, via which FAM201A was found to indirectly regulate the expression of messenger RNA (mRNA) by sponging its targeted microRNAs (miRNAs) [10, 14, 15]. Without lncRNA-directed sponging effects, miRNAs, a class of small noncoding RNAs with 18-24 nucleotides in length that are endogenous and evolutionarily conserved, can destabilize mRNAs or inhibit translation, thereby repressing mRNA expression by complementarily binding to the 3'-untranslated regions of the mRNAs [16, 17].

A large number of miRNAs have been implicated in CC-associated ceRNA networks. For instance, MiR-1271-5p expression was previously reported to be aberrantly downregulated in acute myeloid leukemia [18], colon cancer [19], multiple myeloma [20], and LUAD [21], indicating that it played a tumor-suppressive role in the progression of these cancers through related ceRNA networks. Meanwhile, upregulated miR-1271-5p expression was shown to induce oncogenic effects and associated with unfavorable prognoses in hepatocellular carcinoma (HCC) [22]. However, whether FAM201A regulates miR-1271-5p through the ceRNA network and thus participates in CC progression remains unconfirmed.

In this study, we investigated the effects of FAM201A in CC progression using bioinformatics tools and determined the potential miR-1271-5p-targeted mRNA for identifying a FAM201A-miR-1271-5p-mRNA ceRNA regulatory network in CC, with the hope to propose an original molecular therapy for CC.

## 2. Materials and Methods

### 2.1. Ethics Statement

Written informed consent was obtained from all human participants. All animal experiments were performed following the guidelines of the China Council on Animal Care and Use [23]. The human and animal studies were approved by the Ethics Committee and the Committee of Experimental Animals of Nanfang Hospital (approval number: GD202004020/GD202007027), respectively.

### 2.2. Clinical Sample

CC tissues (*n* = 33) and adjacent normal tissues ($\underset{¯}{n}=33$) were collected during surgical operation at the Second Hospital of Shanxi Medical University in 2020 from CC patients without preoperative chemotherapy, radiotherapy, or immunotherapy. Fresh samples were immediately frozen in liquid nitrogen and stored at -80°C.

### 2.3. Cell Culture

Human cervical endometrial epithelial cells (HCerEpiC; CP-H058, Procell Life Science&Technology Co., Ltd, Wuhan, China) were cultured in EpiLife Media (MEPI500CA, ThermoFisher, Waltham, MA, USA) to reach a confluence around 75% within 10–14 days. CC cell lines, including HeLa (CCL-2), C33a (HTB-31), SiHa (HTB-35), and ME180 (HTB-33) purchased from American Type Culture Collection (ATCC, Manassas, VA, USA), were cultivated in high-glucose Dulbecco's Modified Eagle Medium complete media (DMEM; 11965092, ThermoFisher, USA) supplemented with 2 mM L-glutamine (25030081, ThermoFisher, USA), 10% Fetal Bovine Serum (FBS; 16140071, ThermoFisher, USA), and 1% penicillin-streptomycin (V900929, Sigma-Aldrich, St. Louis, MO, USA) at 37°C with 5% CO_2_.

### 2.4. General/Fluorescent *in Situ* Hybridization

The expression and subcellular location of FAM201A were determined by General/Fluorescent *in situ* hybridization using Digoxigenin-labeled Probe Detection kits (Boster Biological Technology, Wuhan, China). As per the manufacturer's instructions, CC tissues and adjacent normal tissues were fixed in 4% paraformaldehyde (16005, Sigma-Aldrich, USA), dehydrated by ethanol, and transparentized by xylene (95682, Sigma-Aldrich, USA). Then, the tissues were embedded in paraffin (1496904, Sigma-Aldrich, USA) and cut into 4 *μ*m-thick sections, following which the sections underwent dewaxation with xylene and rehydration by ethanol. SiHa and ME180 cells were cultured to reach a concentration of 1 × 10^5^ cells/mL and fixed in 4% paraformaldehyde for 4 hours (h). Afterward, the sections and cells were treated with a standard prehybridization buffer at 68°C for 20 h. Digoxigenin-labeled DNA probes complementary to FAM201A were denaturalized via boiling water bath for 10 minutes (min) and added into the standard prehybridization buffer to formulate prehybridization buffer. The prehybridization buffer was then incubated with the tissues and cells at 68°C for another 20 h. After washing using Wash Solution I, a biotin-labeled anti-Digoxigenin antibody was added to the tissues, followed by the 3,3'-Diaminobenzidine (DAB) treatment for the color-development of the tissues. The cells were supplemented with anti-Digoxigenin antibody (ab420, Abcam, Cambridge, MA, USA) and incubated with Goat anti-mouse IgG H&L (ab150115, Abcam, USA). The nuclei of the cells were dyed using 4',6-diamidino-2-phenylindole (DAPI; D21490, ThermoFisher, USA). Color and fluorescent color signals were observed by a confocal microscope (Raman DXR™3, ThermoFisher, USA) at the magnification of ×200.

### 2.5. Cell Transfection

The pcDNA™3.1/Hygro(+) mammalian expression vectors were used to construct overexpression plasmids of FAM201A and FLOT1, and the empty vector was set as negative control (NC). MiR-1271-5p mimic/mimic control (MC) (miR10005796-1-5/miR1N0000001-1-5) was purchased from RIBOBIO (Guangzhou, China). C33a or ME180 cells (4 × 10^4^) were seeded in 96 well plates until 80% confluence was reached. Transfection working solutions (0.15 *μ*L) were prepared by mixing Lipofectamine 3000 transfection reagents (L3000015, ThermoFisher, USA) and Opti-MEM media (31985062, ThermoFisher, USA). Subsequently, the above plasmids were (2 *μ*g) added into Opti-MEM media (10 *μ*L) together with a P3000 reagent (0.4 *μ*L). Next, the processed plasmids were mixed with the transfection working solution at a ratio of 1 : 1 to obtain an RNA-lipid complex, of which 10 *μ*L of the complex mixture was incubated with the cells at 37°C for 24 h or 48 h.

### 2.6. Cell Counting Kit- (CCK-) 8 Assay

The viability of SiHa or ME180 cells was evaluated using a CCK-8 kit (96992, Sigma-Aldrich, USA). After transfection with FAM201A/FLOT1 overexpression plasmids or miR-1271-5p mimic alone or in combination, SiHa or ME180 cells were seeded into 96-well plates at a density of l × l0^4^ cells/well supplemented with complete media and cultured. The cells in each well were treated with the CCK-8 reagent (10 *μ*L) and incubated for 4 h, at 24-, 48- and 72-h post-transfection. The optical density at a wavelength of 450 nm was determined by a microplate reader (ELx808, BioTek, Winooski, VT, USA).

### 2.7. Bioinformatics Analysis

The targeting relation between FLOT1 and miR-1271-5p was predicted by Targetscan (http://www.targetscan.org/vert_71/).

### 2.8. Dual-Luciferases Reporter Assay

Dual-Luciferase Reporter Assay System (E1910, Promega, Madison, WI, USA) was used to verify the targeting relationships between FAM201A and miR-1271-5p and between miR-1271-5p and FLOT1. SiHa or ME180 cells (4 × 10^4^) were then cultured to attain 70% confluence. Sequences of wild type FLOT1 (WT) (5′-CCCCTCATCUCTCCTTGCCAAAT-3′) and mutant FLOT1 (MUT) (5′-CCCCTCATCUCTCCTGGACAAAT-3′) were cloned onto pMirGLO luciferase vectors (50 ng, E1330, Promega, USA). The cells were cotransfected with the pMirGLO cloned with FLOT1-WT or FLOT1-MUT (2 *μ*g) and miR-1271-5p mimic (2 *μ*g) using Lipofectamine 3000 transfection reagent for 48 h. After cotransfection, the cells were lysed by diluted Lysis Buffer (50 *μ*L, 16189, ThermoFisher, USA) and added with Luciferase Assay Reagent II (100 *μ*L). The activity of firefly luciferase, which was normalized to that of *Renilla* luciferase, was measured using a luminometer (GloMax®20/20, Promega, USA).

### 2.9. Transwell Assay

Transwell chambers (3428, Corning, Corning, NY, USA) were used to assess the migratory and invasive abilities of SiHa cells and ME180 cells after transfection with FAM201A/FLOT1 overexpression plasmids or miR-1271-5p M alone or in combination. The upper chamber was precoated by Matrigel (dilution: 1 : 3; 356234, Corning, USA) for cell invasion assay, while that without Matrigel was used for cell migration assay. The cells were cultured to prepare a cell suspension at a concentration of 5 × 10^5^ cells/ml. Then, 100 *μ*L of the cell suspension was poured into the upper chamber, and 600 *μ*L of DMEM containing 10% FBS was added to the lower chamber. The whole Transwell set was incubated at 37°C for 24 h. Later, the lower chamber was washed twice with phosphate-buffered saline (P5493, Sigma-Aldrich, USA), fixed with 4% paraformaldehyde (P6148 Sigma-Aldrich, USA) and stained with 800 *μ*L Giemsa (10092013, ThermoFisher, USA). After removing nonmigratory or noninvading cells, the remaining cells were observed under × 200 magnification using an inverted microscope (IX71; Olympus, Tokyo, Japan). Cells in five randomly selected fields were counted using ImageJ software and cell migration and invasion rates were calculated.

### 2.10. Murine Xenograft Assay

BALB/c nude mice (Male, 5–6-week-old) were purchased from the Vital River Laboratories (Beijing, China). The mice were maintained under a specific condition (22~ 24°C, 50% humidity, a 12 h:12 h circadian cycle), with free access to a standard mice chow and water. Then, the mice were randomized into four groups (*n* = 6 per group): NC+MC group, FAM201A+MC group, NC+miR-1271-5p M group, and FAM201A+miR-1271-5p M group. After transfection, SiHa cells (5 × 10^6^) with stable expressions of FAM201A, miR-1271-5p or both were subcutaneously injected into the posterior flank of the mice. The size of subcutaneous xenografts (length and width) was measured by a caliper every 7 days, with 5 times in total, and the volume of the xenografts was calculated according to the formula: 0.5 × length × width^2^. Five weeks after the injection, the mice were sacrificed via spinal dislocation under anesthetization using pentobarbital sodium (P010, Sigma-Aldrich, USA), following which the subcutaneous xenografts were resected and weighed.

### 2.11. Quantitative Reverse Transcription Polymerase Chain Reaction (qRT-PCR)

Total mRNAs and miRNAs from CC cell lines and HCerEpiC, as well as CC tissues and the adjacent normal tissues, were extracted by TRIzol lysis buffer (15596018, ThermoFisher) and Small RNA kits (9753Q, TaKaRa, Liaoning, China), respectively. Chloroform (48520-U, Sigma-Aldrich, USA) was used to extract the lysate of mRNA and miRNA. The extracted lysate was centrifuged (12000 × g) at 4°C for 15 min. Then, isopropanol (W292907, Sigma-Aldrich, USA) was applied to precipitate the lysate from water layers via centrifugation (12000 × g) at 4°C for 10 min, which was then washed with 75% ethanol (32205, Sigma-Aldrich, USA) and then isolated from the supernatant. Next, it was resuspended and centrifugated (7500 × g) at 4°C for 10 min and dissolved in 20 *μ*L diethyl pyrocarbonate (DEPC; 40718, Sigma-Aldrich, USA). First-strand cDNAs of the isolated mRNA and miRNA were synthesized using a Synthesis Kit (K1621, ThermoFisher, USA). qPCR was performed on an Applied Biosystems 7500 FAST real-time PCR machine (Applied Biosystems, Foster City, CA, USA) with TB Green® Premix Ex Taq II (Tli RNaseH Plus, RR820Q, TAKARA, China). The primers used are shown in Table 1. The thermocycling conditions were set as follows: 95°C for 10 min, followed by 40 cycles of 95°C for 15 s, and 60°C for 1 min. The expressions of relative genes normalized to U6 or GAPDH were calculated using the 2^−ΔΔCT^ method [24].

### 2.12. Western Blot Analysis

RIPA Lysis and Extraction Buffer (89901, ThermoFisher, USA) was used to harvest total protein from SiHa cells and ME180 cells. The protein concentration was quantitated by the bicinchoninic acid (BCA) Protein Assay Kits (23227, ThermoFisher, USA). The protein (40 *μ*g) and marker (4 *μ*L) (PR1910, Solarbio, Beijing, China) were separated by 10%-12% sodium dodecyl sulfate-polyacrylamide gel electrophoresis (SDS-PAGE) gels (P0670, P0672, Beyotime, Shanghai, China) and laid onto polyvinylidene fluoride (PVDF) membranes (FFP28, Beyotime, China). Afterward, the membranes were blocked with 5% skim milk in Tris Buffered Saline and Tween 20 (TA-999-TT, ThermoFisher, USA) at room temperature for 1 h. Primary antibodies against FLOT1 (ab133497, 47 kDa, 1 : 10000, Abcam, USA), matrix metalloproteinase (MMP)-9 (ab73734, 78 kDa, 1 : 1000, Abcam, USA), MMP-2 (ab37150, 72 kDa, 1 : 1000, Abcam, USA), E-cadherin (ab40772, 97 kDa, 1 : 10000, Abcam, USA), N-cadherin (ab18203, 130 kDa, 1 *μ*g/ml, Abcam, USA), Wnt1 (ab15251, 41 kDa, 1 : 1000, Abcam, USA), *β*-catenin (ab16051, 95 kDa, 1 : 1000, Abcam, USA), p-*β*-catenin (ab27798, 92 kDa, 1 : 500, Abcam, USA), and GAPDH (ab8245, 36 kDa, 1 : 1000, Abcam, USA) were incubated with the membranes at 4°C overnight. Then, secondary antibodies, including Goat Anti-Rabbit IgG (ab205718, 1 : 2000, Abcam, USA) and Goat Anti-Mouse IgG (ab6789, 1 : 2000, Abcam, USA), were incubated with the membranes. The obtained protein was photo-developed using Enhanced Chemiluminescent (ECL) Substrate Reagent Kit (WP20005, ThermoFisher, USA) on an imaging system (iBright CL1500, ThermoFisher, USA). Analysis of the gray value of protein bands was conducted using the ImageJ software (version. 1.52 s, National Institutes of Health, Bethesda, MA, USA).

### 2.13. Statistical Analysis

Measurement data with normal distribution were expressed as mean ± standard deviation (SD). All the experiments were conducted in triplicate. SPSS software (version 21.0, SPSS Inc., Chicago, IL, USA) was used for statistical analysis. The differences between CC tissues and the adjacent normal tissues were analyzed by paired *t*-test. Comparison between the other two groups was performed by independent *t*-test, and those between multiple groups were conducted by one-way analysis of variance (ANOVA) followed by Dunnett's or Turkey's post-hoc test. Pearson's correlation tests were used to analyze the correlation between FAM201A and miR-1271-5p in CC tissues. Statistics with *P* < 0.05 were considered statistically significant.

## 3. Results

### 3.1. FAM201A Was Highly Expressed in CC, Enriched in CC Cell Cytoplasm, and Its Expression Was Negatively Correlated with miR-1271-5p

**Figure 1:**
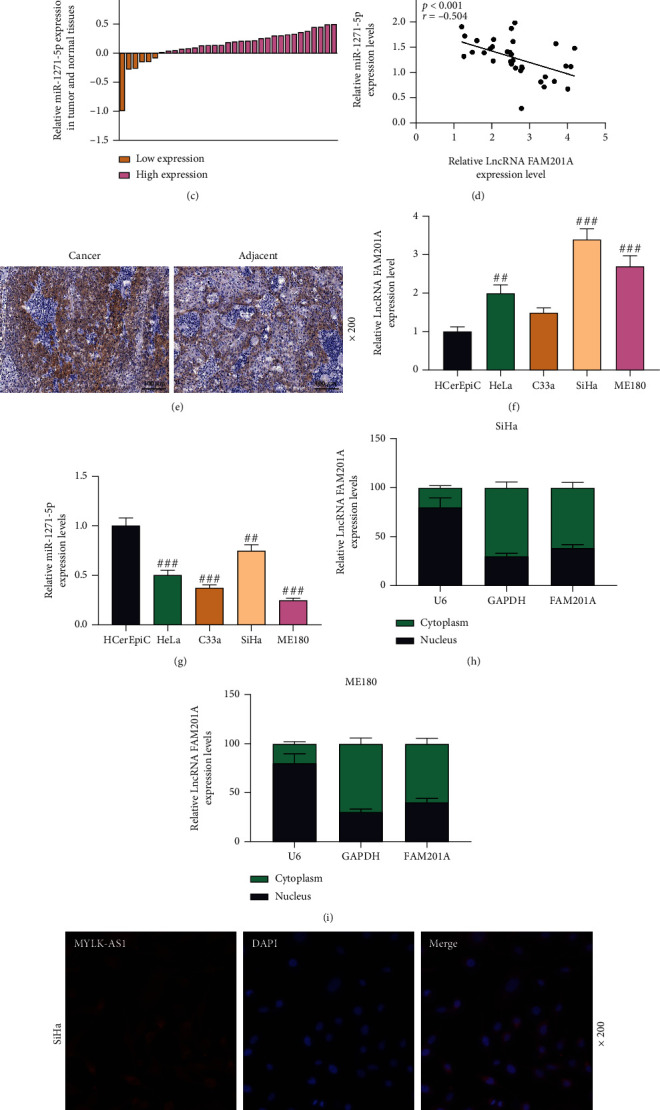
FAM201A was highly expressed in CC and enriched in CC cell cytoplasm, and its expression was negatively correlated with miR-1271-5p. (a) The expression of FAM201A was analyzed in the TCGA-CESC database, including CC samples (*n* = 306) and normal samples (*n* = 13) by GEPIA. (b, c, f, g) The expressions of FAM201A (b, f) and miR-1271-5p (c, g) in CC tissues and the adjacent normal tissues (b, c) as well as in HeLa, C33a, SiHa, ME180 and human endocervical epithelial HCerEpiC cells (f, g) were analyzed by qRT-PCR. (d) The correlation between FAM201A and miR-1271-5p in CC tissues was analyzed by Pearson's correlation tests. (e) The expression of FAM201A in CC tissues and the adjacent normal tissues was assessed by fluorescence *in situ* hybridization (magnification: ×200; scale: 100 *μ*m). (h, i) The subcellular localization of FAM201A was determined by qRT-PCR. (j, k) The expression of FAM201A in SiHa cells and ME180 cells was evaluated by fluorescent *in situ* hybridization (magnification: ×200; scale: 100 *μ*m). ^##^*P* < 0.01; ^###^*P* < 0.001; ^#^ vs. HCerEpiC. CC: cervical cancer; qRT-PCR: quantitative reverse transcription polymerase chain reaction; NC: negative control; FAM201A: the family with sequence similarity 201-member A; M: miR-1271-5p mimic; MC: mimic control; GEPIA: Gene Expression Profiling Interactive Analysis; TCGA-CESC: Cancer Genome Atlas Cervical Squamous Cell Carcinoma and Endocervical Adenocarcinoma; qRT-PCR: quantitative reverse transcription polymerase chain reaction.

According to Gene Expression Profiling Interactive Analysis (GEPIA) based on Cancer Genome Atlas Cervical Squamous Cell Carcinoma and Endocervical Adenocarcinoma (TCGA-CESC) database, FAM201A expression level was higher in CC tissues than that in normal tissues (*P* < 0.05; Figure 1(a)). Then, we harvested 33 pairs of clinical samples including CC tissues and the adjacent normal tissues for the examination of FAM201A expression. The results showed that FAM201A was highly expressed in CC tissues compared with adjacent normal tissues (Figure 1(b)). Considering that miR-1271-5p has been widely reported as a regulator of tumor growth, in this present study, miR-1271-5p expression was downregulated CC clinical samples and compared with adjacent normal tissues (Figure 1(c)), which showed a negative correlation between miR-1271-5p and lncRNA FAM201A via Pearson's correlation analysis (Figure 1(d)). Meanwhile, fluorescence *in situ* hybridization assay confirmed that FAM201A was highly expressed in CC tissues compared with adjacent normal tissues (Figure 1(e)). Moreover, in comparison with HCerEpiC, FAM201A expression level was also highly expressed in CC (HeLa, C33a, SiHa, and ME180) cells, while miR-1271-5p expression was decreased (*P* < 0.01; Figures 1(f) and 1(g)). A relatively higher expression level of FAM201A was seen in SiHa cells and ME180 cells than in other cells above. Therefore, to investigate the role of FAM201A during the progression of CC, SiHa cells and ME180 cells were chosen as cell models in the following related experiments to achieve obvious overexpression of FAM201A. Subsequently, qRT-PCR and fluorescent *in situ* hybridization were performed to determine the subcellular localization of FAM201A. As shown in Figures 1(h) and 1(i) (qRT-PCR) and Figures 1(j) and 1(k) (fluorescent *in situ* hybridization), FAM201A was abundantly expressed in the cytoplasm rather than in the nucleus, suggesting a role of FAM201A in post-transcriptional regulation.

### 3.2. FAM201A Overexpression Increased the Cell Viability, Migration, Invasion and Tumorigenesis of CC *In Vivo* by Downregulating miR-1271-5p Expression

**Figure 2:**
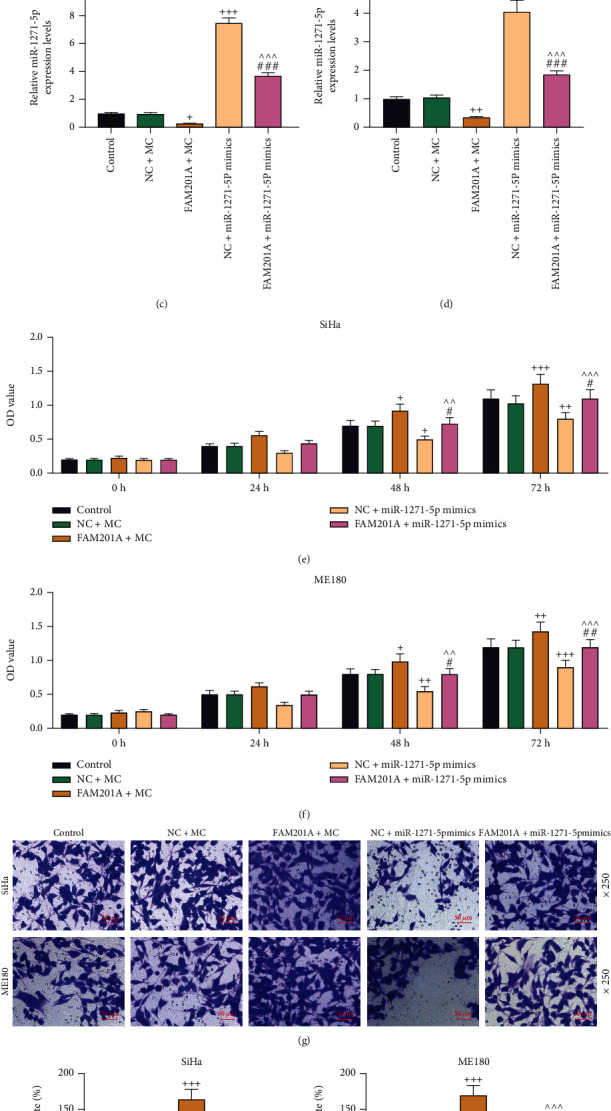
FAM201A overexpression increased CC cell viability and promoted migration by downregulating miR-1271-5p expression. (a-d) The expressions of FAM201A (a, b) and miR-1271-5p (C/D) in CC (SiHa and ME180) cells transfected with FAM201A overexpression plasmids or miR-1271-5p mimic alone or in combination were analyzed by qRT-PCR. (e, f) The viability of CC (SiHa and ME180) cells after transfection with FAM201A overexpression plasmids or miR-1271-5p mimic alone or in combination was measured by CCK-8 assay at 24-, 48-, and 72-h post-transfection. (g-i) The migration of CC (SiHa and ME180) cells after transfection with FAM201A overexpression plasmids or miR-1271-5p mimic alone or in combination was evaluated by the Transwell assay (magnification: ×250; scale: 50 *μ*m). ^+^*P* or ^#^*P* < 0.05; ^++^*P* or ^^^^*Por*^##^*P* < 0.01; ^+++^*P* or ^^^^^*P* or ^###^*P* < 0.001; ^+^ vs. NC+MC; ^^^*vs*. FAM201A+MC; ^#^ vs. NC+miR-1271-5p M. CC: cervical cancer; qRT-PCR: quantitative reverse transcription polymerase chain reaction; CCK-8: cell counting kit-8; NC: negative control; FAM201A: the family with sequence similarity 201-member A; M: miR-1271-5p mimic; MC: mimic control.

**Figure 3:**
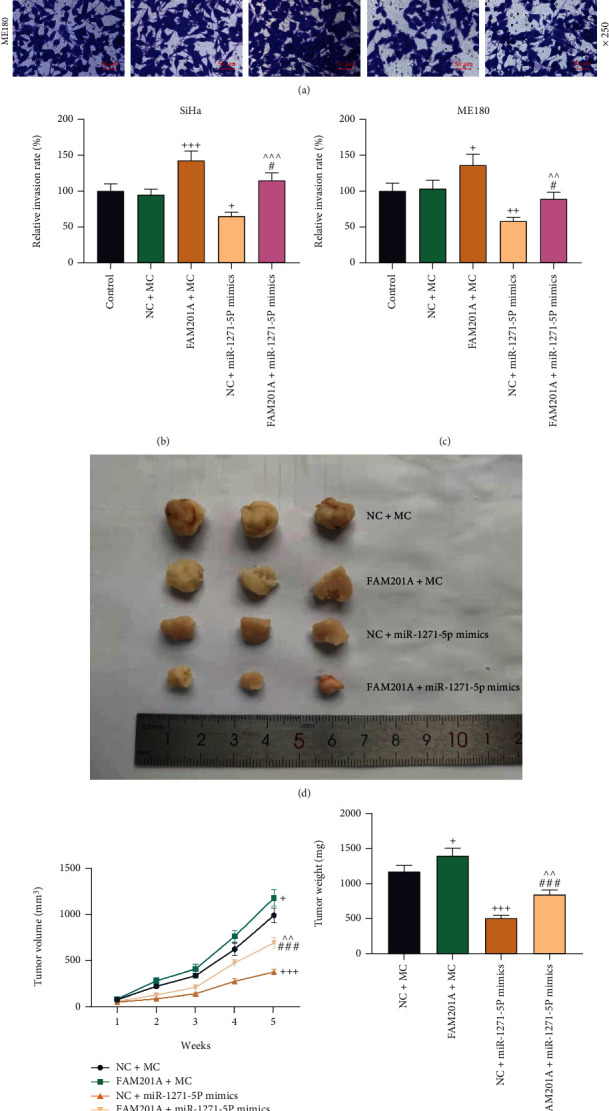
FAM201A overexpression promoted CC cell invasion and CC tumorigenesis *in vivo* by downregulating miR-1271-5p expression. (a-c) The invasion of CC (SiHa and ME180) cells transfected with FAM201A overexpression plasmids or miR-1271-5p mimic alone or in combination was evaluated by the Transwell assay (magnification: ×250; scale: 50 *μ*m). (d) Pictures of subcutaneous xenografts formed by SiHa cells with stable overexpressed FAM201A, miR-1271-5p or both (e, f). The volume (e) and weight (f) of subcutaneous xenografts formed by SiHa cells with stable overexpressed FAM201A, miR-1271-5p or both were measured weekly (e) or at the fifth week after resection (f). ^+^*P* or ^#^*P* < 0.05; ^++^*P* or ^^^^*P* < 0.01; ^+++^*P* or ^^^^^*P* or ^###^*P*;^+^ vs. NC+MC; ^^^ vs. FAM201A+MC; ^#^ vs. NC+miR-1271-5p M. CC: cervical cancer; qRT-PCR: quantitative reverse transcription polymerase chain reaction; NC: negative control; FAM201A: the family with sequence similarity 201-member A; M: miR-1271-5p mimic; MC: mimic control.

Here, functional experiments, including CCK-8, qRT-PCR, Transwell, and murine xenograft assays, were performed. Prior to these assays, the transfection efficiency of FAM201A overexpression plasmids and miR-1271-5p mimic was validated by qRT-PCR, through which we observed that the transfection of both FAM201A overexpression plasmid and miR-1271-5p mimic induced the upregulation of FAM201A and miR-1271-5p expression, respectively (*P* < 0.001; Figures 2(a) and 2(c)). Moreover, upregulation of miR-1271-5p via miR-1271-5p mimic decreased the level of FAM201A, and likewise, FAM201A overexpression caused a lower level of miR-1271-5p (*P* < 0.05; Figures 2(a)–2(d)). Meanwhile, FAM201A overexpression plasmid and miR-1271-5p mimic counteracted the effect of each other on the expressions of FAM201A and miR-1271-5p (*P* < 0.001; Figures 2(a)–2(d)). Then, assays for FAM201A functional examination were performed. CCK-8 assay revealed that FAM201A overexpression enhanced the viability of CC cells at 24, 48, and 72 h, while miR-1271-5p upregulation decreased the viability of CC cells at 24, 48, and 72 h (*P* < 0.05) (Figures 2(e) and 2(f)). Transwell assay demonstrated that CC cells transfected with FAM201A overexpression plasmid migrated and invaded to a greater extent, while the transfection with miR-1271-5p mimic led to repressed migration and invasion (*P* < 0.01; Figures 2(g)–2(i) and 3(a)–3(c)). In murine xenograft assay, the increased trends of tumor volumes and weight gain were found under the promotion of FAM201A overexpression but inhibited by miR-1271-5p upregulation (*P* < 0.05; Figures 3(e)–3(f)). FAM201A overexpression and miR-1271-5p upregulation mutually reversed the effects of each other (Figures 2(e)–2(i) and 3(a)–3(f)).

Taken together, the above results suggested that FAM201A-directed sponging of miR-1271-5p was associated with CC progression.

### 3.3. FAM201A Sponged miR-1271-5p to Increase the Expression of FLOT1, a Direct Target of miR-1271-5p, in CC Cells

**Figure 4:**
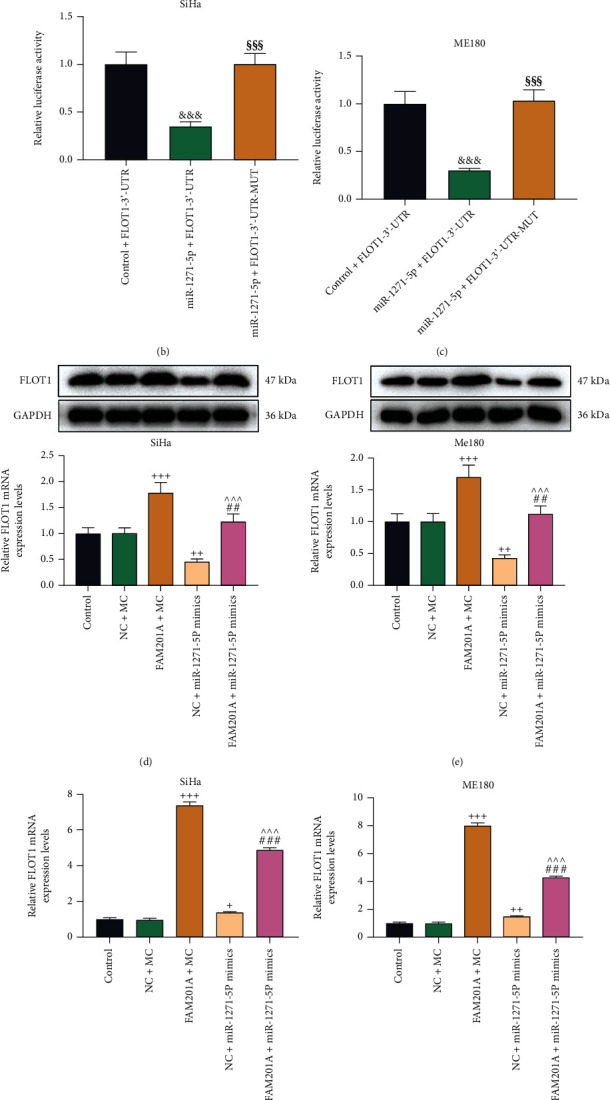
FAM201A sponged miR-1271-5p to increase the expression of FLOT1, a direct target of miR-1271-5p, in CC cells. (a) The targeting relationship between miR-1271-5p and LINC01106 was identified by Targetscan. (b, c) FLOT1 was confirmed as the target of LINC01106 by dual-luciferase reporter assay. (d-g) The expression of FLOT1 in CC (SiHa and ME180) cells after transfection with FAM201A overexpression plasmids or miR-1271-5p mimic alone or in combination was analyzed by qRT-PCR (f, g) and western blot (d, e), with GAPDH as the reference gene. ^+^*P* < 0.05; ^++^*P* or ^##^*P* < 0.01; ^+++^*P* or ^^^^^*P* or ^###^*P* < 0.001; ^+^ vs. NC+MC; ^^^ vs. FAM201A+MC; ^#^ vs. NC+miR-1271-5p M; ^&^ vs. control+ FLOT1-3'-UTR; ^§^vs. miR-1271-5p+FLOT1-3'-UTR. CC: cervical cancer; qRT-PCR: quantitative reverse transcription polymerase chain reaction; NC: negative control; M: miR-1271-5p mimic; MC: mimic control; FLOT1: flotillin 1; 3'-UTR: 3'-untranslated regions.

Prediction using StarBase displayed the complementary binding sites of miR-1271-5p and FLOT1 (Figure 4(a)). Dual-luciferase reporter assay showed that transfection of miR-1271-5p mimic suppressed the luciferase activity of CC cells transfected with vectors inserted with FLOT1-WT (*P* < 0.001), but exerted no obvious effects on the luciferase activity of CC cells transfected with vectors inserted with FLOT1-MUT (Figures 4(b) and 4(c)). Furthermore, FLOT1 expression was found to be upregulated by FAM201A overexpression, but the mRNA and protein levels were knocked down by miR-1271-5p upregulation compared with those in the NC+MC group (*P* < 0.05; Figures 4(d)–4(g)). Also, FAM201A overexpression reversed the miR-1271-5p upregulation-induced FLOT1 knockdown (*P* < 0.01). This trend was further offset by miR-1271-5p upregulation (*P* < 0.001; Figures 4(d)–4(g)).

**Figure 5:**
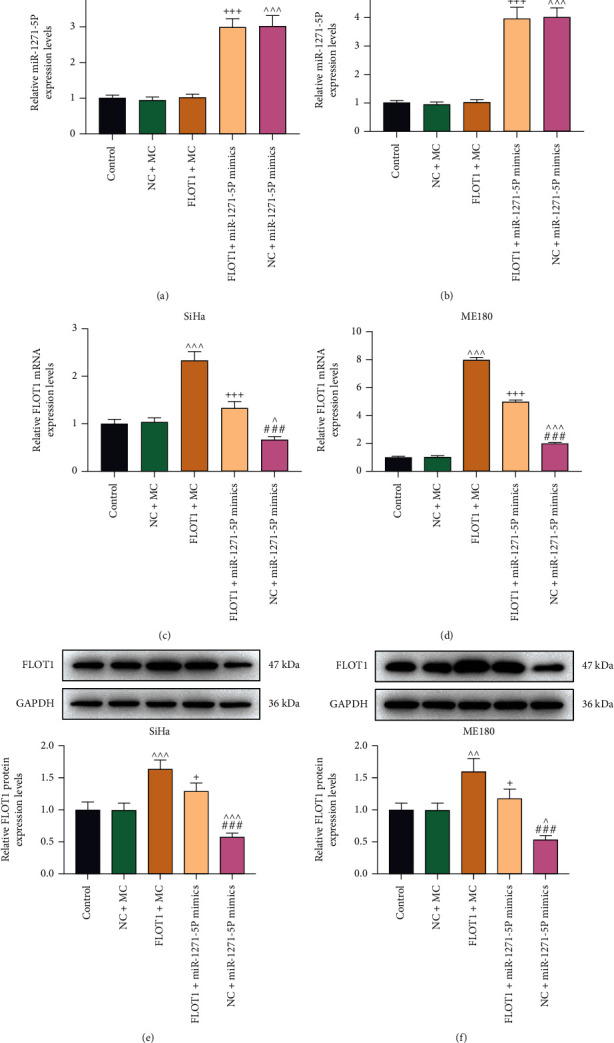
FLOT1 expression was repressed by miR-1271-5p upregulation in CC cells. (a, b, c, d) The expressions of miR-1271-5p (a, b) and FLOT1 (c, d) in CC (SiHa and ME180) cells following transfection with FLOT1 overexpression plasmids or miR-1271-5p mimic alone or in combination were analyzed by qRT-PCR. (e, f) The expression of FLOT1 in CC (SiHa and ME180) cells after transfection with FLOT1 overexpression plasmids or miR-1271-5p mimic alone or in combination was analyzed by western blot, with GAPDH as the reference gene. ^+^*P* or ^^^*P* < 0.05; ^^^^*P* < 0.01; ^+++^*P* or ^^^^^*P* or ^###^*P* < 0.001; ^+^ vs. FLOT1+MC; ^^^ vs. NC+MC; ^#^ vs. FLOT1+miR-1271-5p M. CC: cervical cancer; qRT-PCR: quantitative reverse transcription polymerase chain reaction; NC: negative control; M: miR-1271-5p mimic; MC: mimic control; WT: wild type; MUT: mutant type; FLOT1: flotillin 1.

We observed that no obvious change on miR-1271-5p expression in CC cells after transfection with FLOT1 overexpression plasmid (Figures 5(a) and 5(b)), but CC cells transfected with miR-1271-5p mimic still increased miR-1271-5p expression in cells transfected with the plasmid overexpressing FLOT1 (*P* < 0.001; Figures 5(a) and 5(b)). In addition, compared with miR-1271-5p mimic control, miR-1271-5p upregulation decreased FLOT1 mRNA and protein expressions in negative control-transfected and FLOT1 overexpression plasmid-transfected CC cells, while FLOT1 overexpression plasmid demonstrated the opposite results (*P* < 0.05). Moreover, the effects of miR-1271-5p upregulation and FLOT1 overexpression plasmid were mutually counteractive (*P* < 0.05; Figures 5(c)–5(f)).

### 3.4. FLOT1 Overexpression Resisted miR-1271-5p Upregulation-Induced Decrease in Viability and Inhibition in Migration, Invasion and EMT in CC Cells

**Figure 6:**
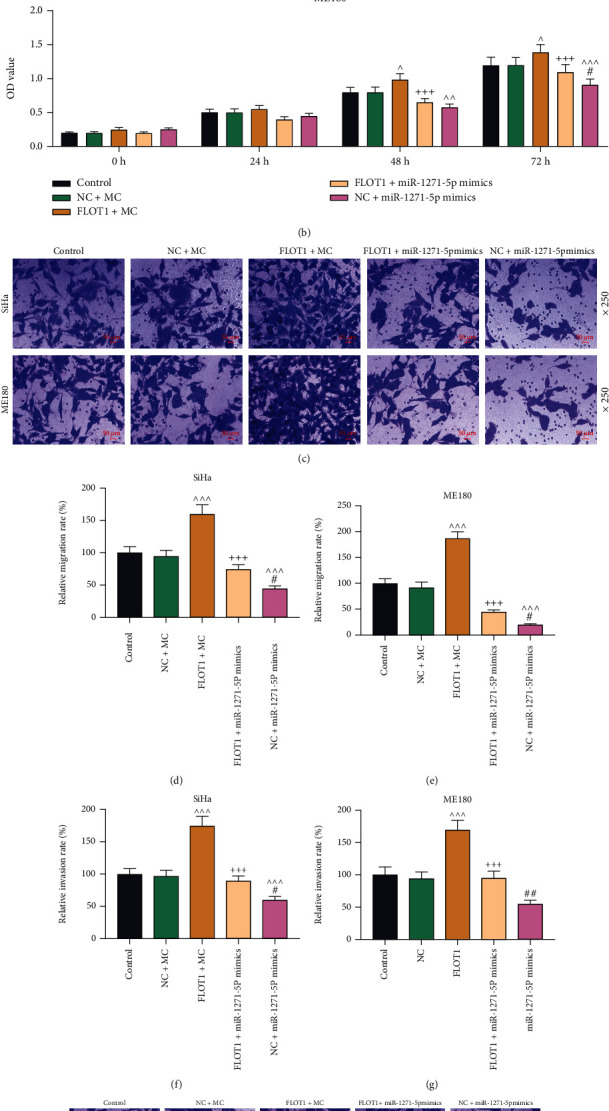
FLOT1 overexpression resisted miR-1271-5p upregulation-induced viability and inhibition in migration and invasion of CC cells. (a, b) The viability of CC (SiHa and ME180) cells transfected with FLOT1 overexpression plasmids or miR-1271-5p mimic alone or in combination was measured by CCK-8 assay at 24-, 48-, and 72-h posttransfection. (c-h). The migration (c-e) and invasion (f-h) of CC (SiHa and ME180) cells after transfection with FLOT1 overexpression plasmids or miR-1271-5p mimic alone or in combination were evaluated by the Transwell assay (magnification: ×250; scale: 50 *μ*m). ^+^*P* or ^^^*P* or ^#^*P* < 0.05; ^++^*P* or ^^^^*P* or ^##^*P* < 0.01; ^+++^*P* or ^^^^^*P* or ^###^*P* < 0.001; ^+^ vs. FLOT1+MC; ^^^ vs. NC+MC; ^#^ vs. FLOT1+miR-1271-5p M. CC: cervical cancer; qRT-PCR: quantitative reverse transcription polymerase chain reaction; CCK-8: cell counting kit-8; NC: negative control; M: miR-1271-5p mimic; MC: mimic control; FLOT1: flotillin 1.

The miRNA-mRNA networks are widely known to regulate CC progression [25]. Here, as FLOT1 was identified as a target mRNA of miR-1271-5p, we investigated FLOT1-miR-1271-5p network-delivered regulation on CC cell phenotypes. The results showed that CC cells transfected with plasmid overexpressing FLOT1 exhibited increased viability at 48 h and 72 h (*P* < 0.05; Figures 6(a) and 6(b)) and more aggressive migration and invasion (*P* < 0.001; Figures 6(c)–6(h)). Additionally, FLOT1 overexpression could counteract miR-1271-5p upregulation-induced effects on the viability of CC cells at 48 h and 72 h (*P* < 0.05), together with their migration and invasion (*P* < 0.05); in turn, miR-1271-5p upregulation also reversed the effects of FLOT1 overexpression on the viability at 48 h and 72 h, migration and invasion of CC cells (*P* < 0.05; Figures 6(a)–6(h)).

**Figure 7:**
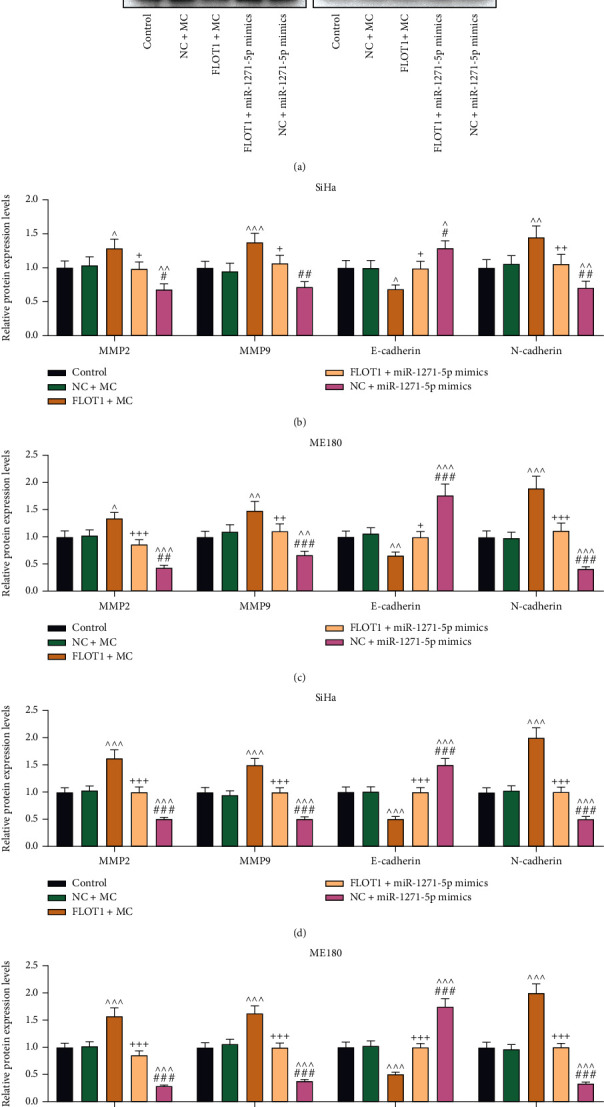
FLOT1 overexpression resisted miR-1271-5p upregulation-induced inhibition of EMT in CC cells. (a-e) The expressions of MMP2, MMP9, E-cadherin and N-cadherin in CC (SiHa and ME180) cells after transfection with FLOT1 overexpression plasmids or miR-1271-5p mimic alone or in combination were analyzed by qRT-PCR (a-c) and western blot (d, e), with GAPDH as the reference gene. ^+^*P* or ^^^*P* or ^#^*P* < 0.05; ^++^*P* or ^^^^*P* or ^##^*P* < 0.01; ^+++^*P* or ^^^^^*P* or ^###^*P* < 0.001; ^+^ vs. FLOT1+MC; ^^^ vs. NC+MC; ^#^ vs. FLOT1+miR-1271-5p M. CC: cervical cancer; qRT-PCR: quantitative reverse transcription polymerase chain reaction; NC: negative control; M: miR-1271-5p mimic; MC: mimic control; FLOT1: flotillin 1; MMP2: matrix metalloproteinase-2; MMP9: matrix metalloproteinase-9.

**Figure 8:**
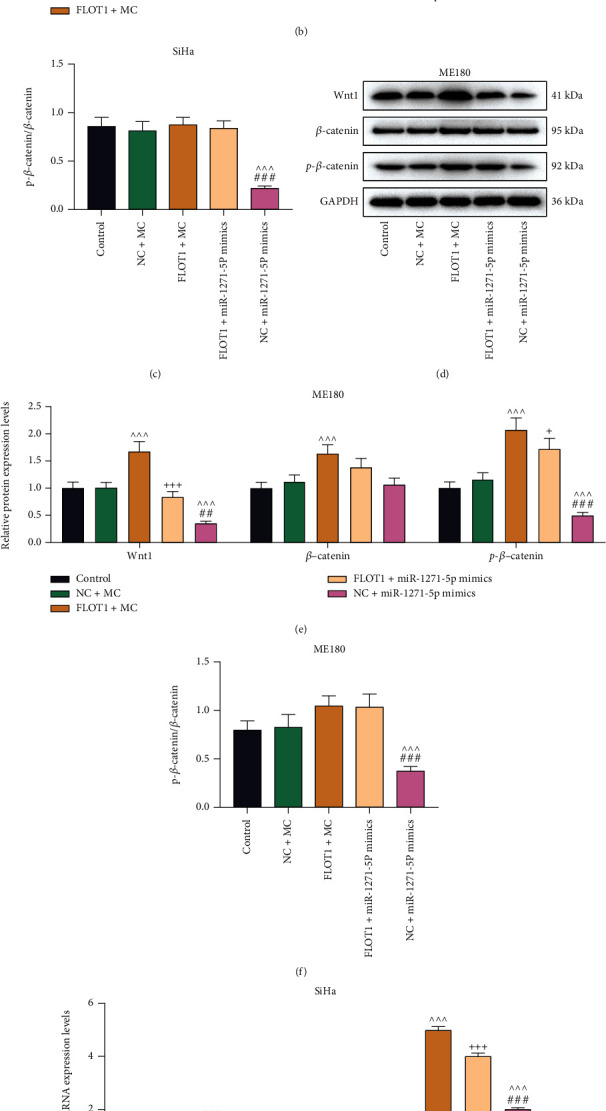
FLOT1 overexpression resisted miR-1271-5p upregulation-induced suppression on the Wnt/*β*-catenin signaling pathway in CC cells. (a-h). The expressions of Wnt1, *β*-catenin, p-*β*-catenin and p-*β*-catenin/*β*-catenin in CC (SiHa and ME180) cells transfected with FLOT1 overexpression plasmids or miR-1271-5p mimic alone or in combination were determined by qRT-PCR (G/H) and western blot (a-f), with GAPDH as the reference gene. ^+^*P* < 0.05; ^++^*P* or ^^^^*P* or ^##^*P* < 0.01; ^+++^*P* or ^^^^^*P* or ^###^*P* < 0.001; ^+^ vs. FLOT1+MC; ^^^ vs. NC+MC; ^#^ vs. FLOT1+miR-1271-5p M. CC: cervical cancer; qRT-PCR: quantitative reverse transcription polymerase chain reaction; NC: negative control; M: miR-1271-5p mimic; MC: mimic control; FLOT1: flotillin 1.

EMT, a biological process, displays distinctive cellular phenotypes and plays vital roles in both cell growth and cancer progression [26]. Thus, the protein and mRNA levels of EMT-related markers were assessed by western blot and qRT-PCR. Both the protein and mRNA levels of MMP-9, MMP-2, and N-cadherin were upregulated by FLOT1 overexpression in CC cells compared with those in the NC+MC group (*P* < 0.05), while compared with those in the NC+MC group, miR-1271-5p upregulation depleted the expressions of these markers (*P* < 0.01) and abolished the FLOT1 overexpression-induced effects on the expressions of these markers (*P* < 0.05) (Figures 7(a)–7(e)). Additionally, the effects of miR-1271-5p upregulation on these EMT-related markers were counteracted by FLOT1 overexpression (*P* < 0.05) (Figures 1(f) and 7(a)). Conversely, E-cadherin, an EMT-related marker, and its protein and mRNA levels were downregulated by FLOT1 overexpression but elevated by miR-1271-5p upregulation (*P* < 0.05), compared with those in the NC + MC group (Figures 8(a)–8(f)). Furthermore, FLOT1 overexpression counteracted the effects of miR-1271-5p upregulation on E-cadherin expression, and miR-1271-5p upregulation also reversed the effects of FLOT1 overexpression (*P* < 0.05) (Figures 8(a)–8(f)).

Collectively, these results indicated that FAM201A sponged miR-1271-5p to induce FLOT1 expression, thereby promoting CC progression.

### 3.5. FLOT1 Overexpression Resisted miR-1271-5p Upregulation-Induced Suppression on the Wnt/*β*-Catenin Signaling Pathway in CC Cells

The Wnt/*β*-catenin signaling pathway, a developmental pathway, is crucial in normal stem cell function and is frequently aberrantly activated in various types of cancer [27–29]. In this research study, the protein and mRNA levels of Wnt1, *β*-catenin and p-*β*-catenin were determined by western blot and qRT-PCR assays. The result showed they were uniformly upregulated by FLOT1 overexpression but downregulated by miR-1271-5p upregulation (*P* < 0.001), compared with those in the NC+MC group (Figures 8(a)–8(e) and 8(g)–8(h)). Moreover, when compared with the NC+MC group, the p-*β*-catenin/*β*-catenin ratio was also depleted by miR-1271-5p upregulation in CC cells but not significantly changed by FLOT1 overexpression (*P* < 0.01) (Figures 8(g) and 8(h)).

Besides, FLOT1 overexpression resisted the miR-1271-5p upregulation-induced inhibitory effect in the protein and mRNA expressions of Wnt1 and *β*-catenin (*P* < 0.01). It also increased the p-*β*-catenin/*β*-catenin ratio in CC cells transfected with miR-1271-5p mimic and the increase in these markers expression levels by FLOT1 overexpression were reversed by miR-1271-5p upregulation (*P* < 0.01) (Figures 8(a)–8(e) and 8(g)–8(h)).

Overall, these results indicated that FAM201A overexpression-mediated miR-1271-5p/FLOT1 axis promoted CC progression by activating the Wnt/*β*-catenin signaling pathway.

## 4. Discussion

In 2003, the World Health Organization considered CC preventable in women [20]. However, due to metastasis, the median survival time of CC patients remains mediocre [30]. Metastasis is a distinctive malignant sign that can be subdivided into two types, hematogenous metastasis and lymphatic metastasis [6, 31], of which lymph metastasis is the leading factor for CC-associated poor prognosis and death [32]. Metastasis in most human cancers implicates both cellular and molecular alterations [33], identifying that the molecular mechanism in CC is very important for hindering the development of metastasis and other malignant phenotypes.

LncRNA-mediated mechanisms have been widely unveiled in the carcinogenesis, progression, and therapy resistance of CC [34]. Numerous lncRNAs, including FAM201A, have been confirmed to function as an oncogene in multiple types of human cancers by suppressing malignant phenotypes such as cancer cell proliferation, migration, invasion, and *in vivo* tumorigenesis [10–12]. In line with these studies, our study newly identified FAM201A as a key player in promoting CC carcinogenesis and progression. Analysis of the TCGA-CESC database showed that FAM201A was highly expressed in CC. To increase the credibility of this study's results, CC tissues and cell lines (HeLa, C33a, SiHa, and ME180) were used to assess FAM201A expression. We detected a unanimous increase in FAM201A expression in all CC *in vitro* and *in vivo* samples, which was consistent with the FAM201A expression patterns in TNBC and lung cancer, where FAM201A played oncogenic roles [10–12]. The specific tumor-promoting role of FAM201A was illustrated in previous studies, which reported that knocking down FAM201A led to significantly suppressed proliferation, migration, and invasion of TNBC or LSCC cells [10–12]. In line with the role of FAM201A in TNBC and LSCC, our study discovered a positive association between FAM201A overexpression and the biological behaviors of CC, including cell viability, migration, invasion, and *in vivo* tumorigenesis, which suggested that this oncogenic role of FAM201A also existed in CC.

Functional analyses of FAM201A-miRNA-mRNA ceRNA networks indicated that FAM201A could sponge miRNAs and unleash mRNAs from the binding of FAM201A, with miRNAs critical for the promotion of cancer progression [10]. Our study found that miR-1271-5p was negatively correlated with FAM201A in CC tissues, implying that FAM201A sponged miR-1271-5p in CC. Previous studies reported that miR-1271-5p expression was significantly downregulated and miR-1271-5p exerted a tumor-suppressive effect in several cancers [18, 35]. Preventing oncogene-directed sponging of miR-1271-5p led to the inhibition of cancer progression, as evidenced by Zhang et al. [35], who found that miR-1271-5p upregulation from the knockdown of lncRNA-ZFAS1 constrained *in vitro* development of glioma. In light of Zhang et al.'s evidence, our findings demonstrated that miR-1271-5p upregulation reversed the promotive effect of FAM201A overexpression on the progression of CC, suggesting that FAM201A facilitated the progression of CC by sponging miR-1271-5p.

Furthermore, it was reported that miRNA-mRNA interaction emerged following the interaction between lncRNA and miRNA in ceRNA networks associated with the pathological conditions in cancer [36]. Wang et al. showed that the upregulation of miR-1271-5p by MALAT1 knockdown inhibited the growth and migration of ovarian cancer cells and simultaneously silenced its target mRNA E2F5 [37]. In our study, bioinformatics prediction theoretically identified FLOT1 as the target of miR-1271-5p, which was subsequently validated by our dual-luciferase reporter assay results. Similarly, our findings showed that FAM201A overexpression downregulated miR-1271-5p expression to elevate FLOT1 expression and concomitantly promoted *in vitro* CC progression, indicating that the overexpressed FAM201A-directed ceRNA network with the miR-1271-5p/FLOT1 axis promoted CC progression.

FLOT1, a pivotal marker of lipid rafts that modulates membrane receptor signaling, has been reported to participate in membrane trafficking and affect cell adhesion and invasion, thereby displaying a role in tumorigenesis [38, 39]. The overexpression of FLOT1 has been previously discovered to promote migration and invasion and induce recurrence of bladder transitional cell carcinoma [38], activate oncogenic ALK signaling to drive malignant phenotypes of neuroblastoma [40], and sustain inflammatory signaling to facilitate the growth and invasion of esophageal squamous cell carcinoma cells [40, 41]. For CC, FLOT1 was shown to serve as the downstream target of miR-1294 to form a miR-1294/FLOT1 axis, and its expression can be repressed by the upregulation of miR-1294, thereby inhibiting the progression of CC malignant phenotypes [42, 43]. Likewise, in our *in vitro* experiments, miR-1271-5p upregulation decreased FLOT1 expression and offset FLOT1 overexpression-induced promotion. Meanwhile, FLOT1 overexpression could also counteract the inhibitory effects of miR-1271-5p upregulation on cell viability, migration and invasion. According to these findings, we concluded that targeting the miR-1271-5p/FLOT1 axis could be the underlying mechanism via which FAM201A induced CC progression.

Accumulating evidence indicated that EMT, a hallmark of carcinogenesis, functionally contributed to tumor invasion, migration and metastatic dissemination [44]. The phenotype of EMT mainly involves the transformation of epithelial cells to mesenchymal-like cells, allowing them to invade surrounding tissues [45, 46]. Induction of EMT is accompanied by the loss of epithelial adhesion molecule E-cadherin [47] and an increase in mesenchymal marker N-cadherin [48]. Moreover, during EMT, MMPs, a family of zinc-dependent endoproteases, degrade the extracellular matrix to facilitate EMT [49]. Secretion of MMP-2 and MMP-9 was shown to break down the basement membrane and promote lymph node invasion and cancer metastasis, thus leading to poor prognoses [50, 51]. In this study, we found that E-cadherin levels in CC cells were decreased by FLOT1 overexpression but increased by miR-1271-5p upregulation, and an opposite trend was seen on the levels of N-cadherin, MMP-2, and MMP-9 when FLOT1 was overexpressed or miR-1271-5p expression was upregulated. Besides, we discovered that FLOT1 overexpression counteracted miR-1271-5p upregulation-induced effects on the expressions of these EMT-related markers and vice versa. Collectively, these findings indicated that FAM201A facilitated EMT and promoted CC progression by targeting the miR-1271-5p/FLOT1 axis.

The Wnt/*β*-catenin pathway, which plays an essential role in embryogenesis, homeostasis, and stem cell regeneration and pluripotency, is activated in CC as a promoter of cancer progression [52, 53]. Likewise, our results demonstrated that the levels of Wnt1, *β*-catenin and p-*β*-catenin in CC cells were positively correlated with FLOT1 overexpression, while the levels of these markers were negatively correlated with miR-1271-5p upregulation. Besides, our study revealed that FLOT1 overexpression could also restore the expressions of Wnt1, *β*-catenin, and p-*β*-catenin in CC cells after the upregulation of miR-1271-5p, which indicated that FLOT1 overexpression counteracted the inhibitory effects induced by miR-1271-5p upregulation on the Wnt/*β*-catenin pathway, thus promoting the progression of CC.

Considering that FAM201A was overexpressed in CC cells and acted through the FAM201A-miR-1271-5p-FLOT1 ceRNA network, they might be targeted and used to develop novel potential molecular target to improve CC treatment outcomes, with FAM201A as a potential diagnostic biomarker for CC and possible indicator of FAM201A-targeted treatment for individualized treatment of patients expressing high levels of FAM201A. However, considering limitations such as lack of survival analysis, no assessment to determine the association of FAM201A with pharmacological treatment, and others, these findings should be further verified in translational and clinical studies.

## 5. Conclusion

In conclusion, the current study revealed that FAM201A, which was highly expressed in CC, promoted CC progression via sponging miR-1271-5p to upregulate FLOT1 expression. Moreover, CC progression was also promoted via regulating the miR-1271-5p/FLOT1 axis by activating the Wnt/*β*-catenin pathway. Thus, this study proposed the FAM201A-miR-1271-5p-FLOT1 ceRNA network as an original molecular target for prevention against CC.

## Figures and Tables

**Table 1:** Primers used in quantitative reverse transcription polymerase chain reaction for target genes.

Genes	Species	Forward		Reverse
FAM201A	Human	5′-TCTCTGATGGGAGCCTCTTTA-3′	5′-CAAGCCACAGACGGAGAAA-3′	
miR-1271-5p	Human	5′-CTTGGCACCTAGCAAGCACTCA-3′	5′-GCGAGCACAGAATTAATACGAC-3′	
Flotillin-1	Human	5′-CCATCTCGTCACTGGCATT-3′	5′-CGCCAACATCTCCTTGTTC-3′	
MMP-2	Human	5′-TACAGGATCATTGGCTACACACC-3′	5′- GGTCACATCGCTCCAGACT-3′	
MMP-9	Human	5′-TGTACCGCTATGGTTACACTCG -3′	5′- GGCAGGGACAGTTGCTTCT-3′	
E-cadherin	Human	5′-ATTCTGATTCTGCTGCTCTTG-3′	5′-AGTCCTGGTCCTCTTCTCC-3′	
N-cadherin	Human	5′- AACCTAGCCTACTGGCCAAA -3′	5′- AACATCGAGGTCGTAAACCC-3′	
Wnt1	Human	5′- CGATGGTGGGGTATTGTGAAC-3′-	5′- CCGGATTTTGGCGTATCAGAC-3′	
*β*-Cadherin	Human	5′- GAGCTGCCATGTTCCCTGAG -3′	5′- CAGTTGTCAATTTGATTAAC-3′	
GAPDH	Human	5′-GAGAAGGCTGGGGCTCATTT-3′	5′-AGTGATGGCATGGACTGTGG-3′	
U6	Human	5′-CTCGCTCGGCAGAACA-3′	5′-AACGCTTCACGAATTTGCGT-3′	

## Data Availability

The analyzed data sets generated during the study are available from the corresponding author on reasonable request.
